# Commentary: Achilles' heel

**DOI:** 10.1016/j.xjon.2020.04.006

**Published:** 2020-05-01

**Authors:** Chris C. Cook, Harold G. Roberts, Lawrence M. Wei, Vinay Badhwar

**Affiliations:** Department of Cardiovascular and Thoracic Surgery, West Virginia University, Morgantown, WVa

**Figure:**
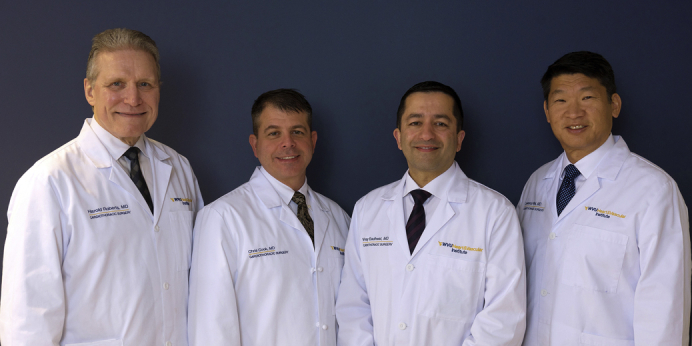
Harold G. Roberts, MD, Chris C. Cook, MD, Vinay Badhwar, MD, and Lawrence M. Wei, MD

**Central Message**

Left ventricular rupture following prosthetic mitral valve replacement might be avoided by valve-sparing techniques and vigilance at the time of debridement to maintain or support annular integrity.

See Article page 48.

David^1^ shares a case series and an erudite summary of the daunting complication of left ventricular (LV) rupture during mitral valve replacement (MVR). David^1^ illustrates the operative recognition and immediate management of 6 patients, all of whom survived. This is particularly laudable because the operative mortality of this complication has been reported to range from 50% to 90%.^2^^,^^3^

Although performing prosthetic MVR is not usual, few surgeons have had the misfortune of having to deal with LV rupture. Bright red blood emanating from the posterior pericardium upon separation from cardiopulmonary bypass following MVR is universally accompanied by a sinking feeling in the operator. Should an ill-prepared surgeon attempt lifting the heart to locate the bleeding source in this setting, the maneuver may prove fatal. As outlined by David,^1^ the key steps in managing this complication are recognizing preoperative risk factors such as mitral annular calcification (Figures 1 and 2), operative prevention, and rapid open operative correction.

**Figure 1:**
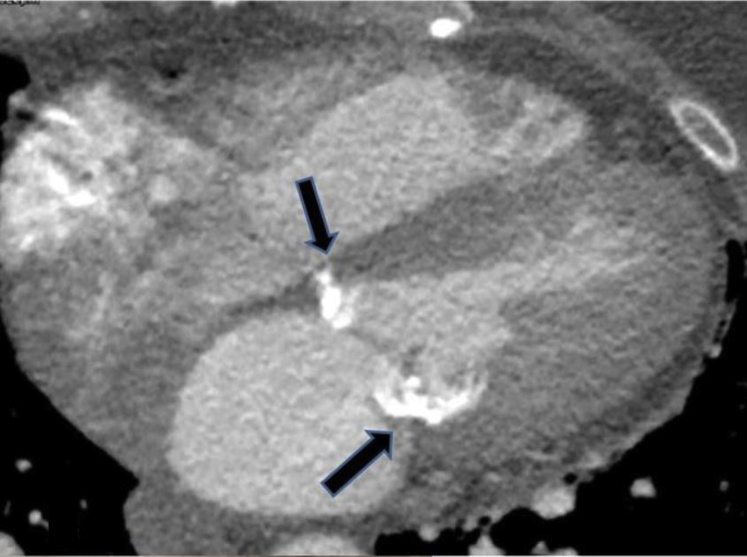
Mitral annular calcification with deep ventricular extension (*arrows*) can be identified on preoperative computed tomography.

**Figure 2:**
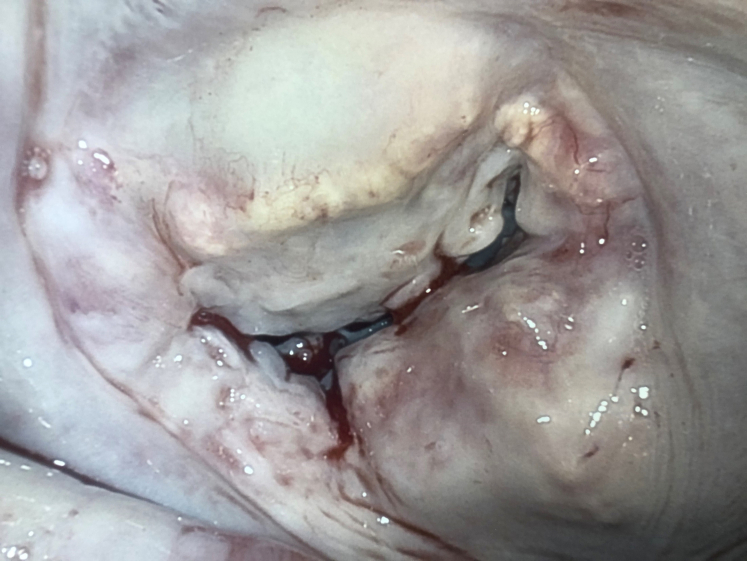
When operative exposure reveals loss of annular definition and deep atrial and ventricular involvement, surgeons should consider the patient at high risk for postoperative left ventricular rupture.

Predispositions to this complication include female patients, a small LV cavity, advanced age, severe mitral annular calcification, and implantation of higher-profile bioprosthetic valves. Vigilance and patch correction at the time of annular debridement may avoid type 1 LV disruption, whereas avoiding deep debridement involving the papillary muscles can mitigate type 2 LV disruption. Posterior leaflet and total leaflet sparing MVR operations have nearly eliminated this complication.^4^^,^^5^ In the presence of predisposing factors and operative concern of postdebridement annular integrity, concomitant oversized annular patching with autologous pericardium or similar substitute may prevent LV rupture following MVR.^6^ Should clinical presumption of LV injury occur with postbypass ejection, rapid decision making as outlined by David^1^ is required and removal of the prosthesis is recommended along with placement of a large, oversized patch without tension before re-replacement. Attempts at epicardial solutions with sealants have not proven to be uniformly effective. These few maneuvers are the essential steps to the prevention and management of this otherwise potentially lethal complication of MVR.

Greek mythological reference to Achilles' heel symbolizes that despite overall strength, a focal vulnerability may lead to downfall. Surgeons are well versed in the reproducible techniques of prosthetic MVR. Valve-sparing methods and adaptive strategies to address mitral annular calcification are the established necessary standards to avoid this potential vulnerability and circumvent this pitfall. Vigilance can often save patients from this often fatal problem.

## References

[1] David T. (2020). Left ventricular rupture after mitral valve replacement. J Thorac Cardiovasc Surg Open.

[2] Otaki M., Kitamura N. (1993). Left ventricular rupture following mitral valve replacement. Chest.

[3] Zhang H.J., Ma W.G., Xu J.P., Hu S.S., Zhu X.D. (2006). Left ventricular rupture after mitral valve replacement: a report of 13 cases. Asian Cardiovasc Thorac Ann.

[4] Spencer F.C., Galloway A.C., Colvin S.B. (1985). A clinical evaluation of the hypothesis that rupture of the left ventricle following mitral valve replacement can be prevented by preservation of the chordae of the mural leaflet. Ann Surg.

[5] Guo Y., He S., Wang T., Chen Z., Shu Y. (2019). Comparison of modified total leaflet preservation, posterior leaflet preservation, and no leaflet preservation techniques in mitral valve replacement—a retrospective study. J Cardiothorac Surg.

[6] Kim S.W., Jeong D.S., Sung K., Kim W.S., Lee Y.T., Park P.W. (2018). Surgical outcomes of mitral valve replacement with concomitant mitral annular reconstruction. J Card Surg.

